# Dicing of composite substrate for thin film AlGaInP power LEDs by wet etching

**DOI:** 10.1038/s41598-021-90425-x

**Published:** 2021-05-25

**Authors:** Ray-Hua Horng, Shreekant Sinha, Fu-Gow Tarntair, Hsiang-An Feng, Chia-Wei Tu

**Affiliations:** 1 Institute of Electronics, National Yang Ming Chiao Tung University, Hsinchu, 30010 Taiwan, ROC; 2 Center for Emergent Functional Matter Science, National Yang Ming Chiao Tung University, Hsinchu, 30010 Taiwan, ROC; 3 Department of Photonics, National Yang Ming Chiao Tung University, Hsinchu, 30010 Taiwan, ROC; 4 Ingentec Corporation, Zhunan Township, Miaoli County 35059 Taiwan, ROC

**Keywords:** Engineering, Chemical engineering

## Abstract

In this paper, thin film AlGaInP LED chips with a 50 μm thick composite metal substrate (Copper-Invar-Copper; CIC) were obtained by the wet etching process. The pattern of the substrate was done by the backside of the AlGaInP LED/CIC. There was no delamination or cracking phenomenon of the LED epilayer which often occurs by laser or mechanical dicing. The chip area was 1140 μm × 1140 μm and the channel length was 360 μm. The structure of the CIC substrate was a sandwich structure and consisted of Cu as the top and bottom layers, with a thickness of 10 μm, respectively. The middle layer was Invar with a 30% to 70% ratio of Ni and Fe and a total thickness of 30 μm. The chip pattern was successfully obtained by the wet etching process. Concerning the device performance after etching, high-performance LED/CIC chips were obtained. They had a low leakage current, high output power and a low red shift phenomenon as operated at a high injected current. After the development and fabrication of the copper-based composite substrate for N-side up thin-film AlGaInP LED/CIC chips could be diced by wet etching. The superiority of wet etching process for the AlGaInP LED/CIC chips is over that of chips obtained by mechanical or laser dicing.

## Introduction

It is well known that thin film AlGaInP with a mirror substrate created by epilayer transferring can offer the maximum output power for power red light-emitting diode (LED) applications due to the lattice-matching GaAs substrate being an absorbing substrate^1,2^. (Al_x_Ga_1−x_) _0.5_In_0.5_P based red LEDs whose internal quantum efficiencies (IQE) exceed 90% because of epilayer grown on precisely lattice-matched GaAs substrate by MOCVD without the generation of redundance displacement^3–7^. In general, AlGaInP LED epilayers have always been transferred to a CuW substrate or Si permanent substrates with a mirror structure. Nevertheless, a CuW metal substrate is expensive, it is difficult to achieve a thickness below 150 μm, and it is hard to dice by dicing saw and laser. Concerning the Si permanent substrate, it is necessary to perform more processes, such as lapping Si substrate to a 150-μm thickness, depositing the Al metal, and performing thermal annealing (to obtain the Ohmic contact property between the Al metal and Si)^8,9^. If the thickness of Si substrate is too thin (< 150 μm), the cracking phenomenon of the LED epilayer could often occur during the processing. For these kinds of substrates, it is required to dice the wafers into chips using a saw or laser-based dicing^10–14^. Although the diamond saw and laser dicing processes are mature, they require extra time and can easily induce damage, e.g. epilayer delamination or chip cracking. Damage to the LED chips during laser and saw dicing is a serious issue. This technology faces a number of challenges to meet demands for next-generation device manufacturing processes, including chipping and cracking of the dice, limitations in street width, limitations in die shape, mechanical stress of the dies, and limitations in processed materials^15,16^. To overcome these problems, wet chemical etching dicing, which is a novel substrate level dicing method for low cost and high-performance LEDs, was proposed. Wet etching has the potential to dice a whole substrate with thicknesses less than 100 μm. Correspondently, crack could be induced if the Si substrate was thinned less than 150 μm. In contrast, no physical damage to the LED chips during this process (such as cracking) is likely to occur^17–21^. Therefore, the strength of LED chip with CIC substrate can be improved as compared to that of LED with thin Si substrate resulting from saw or laser dicing^22,23^. Concerning the CIC substrate, mechanical strength has been already proposed and fabricated for thin film AlGaInP LED applications in our recent study^8,24^. In this study, the thin film AlGaInP epilayer was successfully transferred to a CIC substrate with a 50-μm thickness using wafer bonding and epilayer transferring technologies^3,25,26^. Thin film AlGaInP LED chips with a composite metal substrate were developed and obtained high-performance LED/CIC chips using the wet etching process. The substrate pattern was performed on the backside of AlGaInP LED/CIC chips with a 1200-μm^2^ chip size and a 300-μm channel width. Moreover, the chip pattern was successfully obtained. The results indicated that dicing could be achieved with low leakage current, high output power, and a low red shift phenomenon by the wet etching process.

## Results and discussion

**Figure 1:**
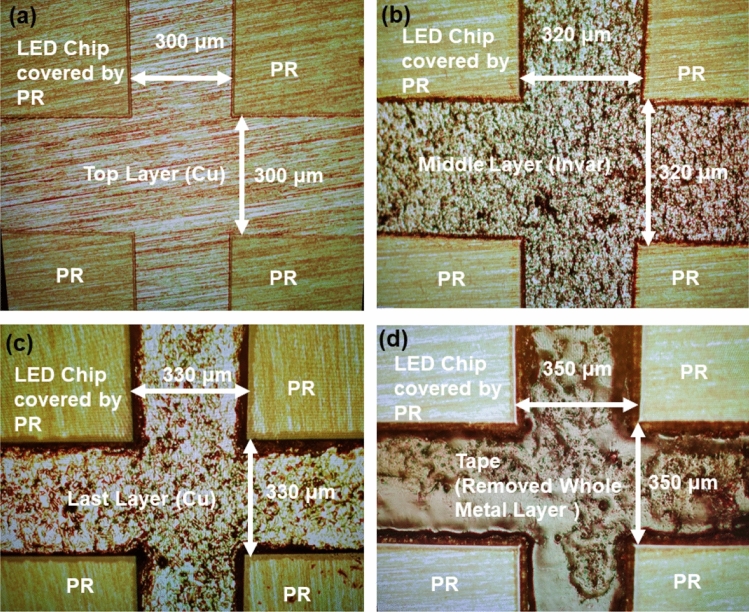
Channel variations of the chip pattern during the dicing process as measured by optical microscope (OM): (**a**) top layer (Cu); (**b**) middle layer (Invar); (**c**) bottom layer (Cu); (**d**) removed whole layer.

It is well known that most chemical etching processes for metal are a type of isotropic etching, and that inside edge etching can exist. To evaluate the existence of side etching, the etching channel was measured before and after the etching process. Because the CIC substrate was a composite metal substrate made of Cu, Invar and Cu layers, the dicing was processed in three steps. Every layer was etched by the chemical solution. It is worthy to mention that both the CuR-8000S and NiE-7520 chemical solutions were used to etch the Cu and Invar layers, respectively. Both presented high metal etching selectivity because the top and bottom layer (Cu) were etched away from the CuR-8000S. Moreover, etching of the top layer (Cu) stopped when the middle layer (Invar) was exposed. The invar layer was removed from the NiE-7520 and the etching stopped when the bottom layer (Cu) was exposed. Figure 1 shows the channel variation of the chip pattern before and after CIC etching, as examined by an optical microscope (OM). As shown in Fig. 1a, the channel dimension defined by photolithography was 300 um. After removing the 10-um thickness of the top layer (Cu) in the channel region by a chemical solution (CuR-8000S) to expose the Invar layer, it was found that the size of the channel became wider by about 20 um, from 300 to 320 um, as shown in Fig. 1b. Approximately seven to eight minutes were needed to etch the top Cu layer. After removing the 30-um thickness of the Invar layer by NiE-7520 to expose the last Cu layer, the channel dimension further increased from 320 to 330 um, as shown in Fig. 1c. The etching time for this step was about 9 to 10 min. Figure 1d shows the OM image after removing the last Cu layer in the channel region. The final dimension of the channel was 350 um. It is worthy to mention that all chips were still attached on the tape. Moreover, the last etched layer took less time (about 4–5 min) and had a high etching rate as compared to the top layer. This resulted in the etching area becoming larger than that of the first Cu layer.

**Figure 2:**
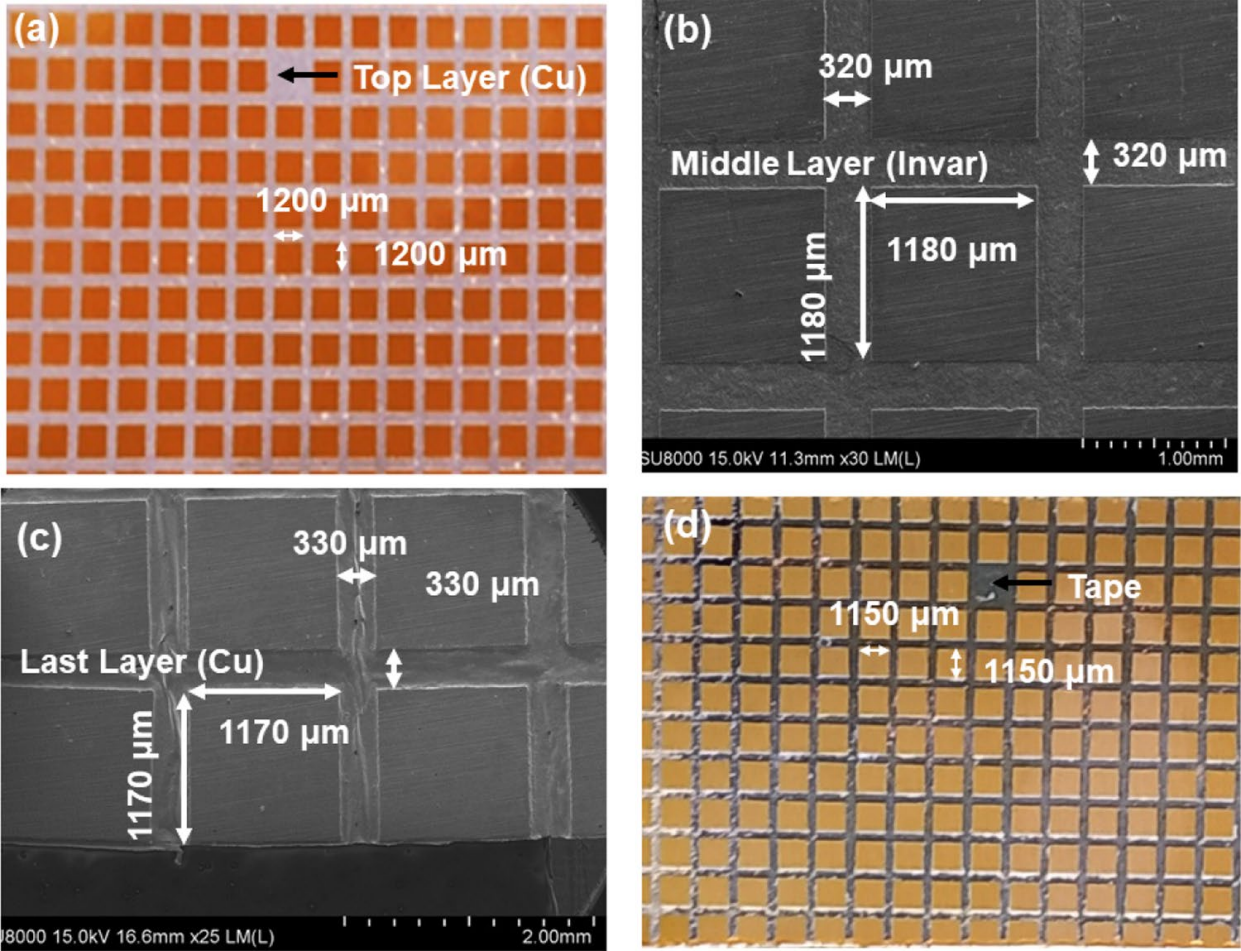
Chip dimensions before and after wet etching from the back side of the CIC for dicing: (**a**) OM image of the chip pattern before the etching process; (**b**) SEM image of the middle layer (Invar); (**c**) SEM image of the last layer (Cu); (**d**) OM image of the diced chip.

The chip dimensions before and after the wet etching process from the backside of the CIC metal substrate was examined by OM and SEM, as shown in Fig. 2. Before etching, the epilayer with the CIC metal substrate was face down and attached to the tape. A double side aligner photolithography machine was used to define a chip area of 1200 um × 1200 um, which was protected by PR, shown in Fig. 2a. After etching the first Cu layer, the chip dimension was measured by SEM and became 1180 um × 1180 um with a channel width of 320 um, as shown in Fig. 2b. This measurement agreed with that measured by OM, shown in Fig. 1b. Concerning the Invar etching, the size of the chips was further reduced by about 10 μm from each side and was reciprocal with the channel width. Figure 2c presents the top-view SEM image of the last Cu layer in the channel region, in which the dimension of the chip was 1170 um × 1170 um. For discrete dicing chips, the last Cu layer was needed to etch away. Figure 2d shows the chips pattern and OM image after removing the last layer (Cu) from the channel region. The size of the chips became 1150 um × 1150 um, after which the LEDs took on a chip form. The dimensions of LED chips changed from 1200 um × 1200 um to 1150 um × 1150 um. Noted that the etching yield is above 99%. The 1% loss was resulted from chips peel off from the tape during etching. A summary of the etching layer parameters for dicing by a chemical solution are shown in Table 1. The etching rate of first Cu is about 1.43–1.25 μm /min. Then the etching rate of Invar is about 3.3–3 μm/min. The etching rate of the last Cu layer is about 2.5–2 μm/min. It is worthy to mention that the lower etch rate for the first Cu layer than that of the second Cu layer, it could be due to the surface being oxidized for the first layer. After the Invar etched away, the Cu is fresh and easily etch by chemical.

**Table 1:** Summary of the etching layers during the dicing process.

Layers	Thickness of layer (μm)	Chip size after etching layers (μm)	Channel width after etching layers (μm)	Etching time (min.)	Etching rate (μm/min)	Side edge etching (μm)
Cu	10	1180	320	7–8	1.43–1.25	20/side
Invar	30	1170	330	9–10	3.3–3	10/side
Cu	10	1150	350	4–5	2.5–2	20/side

Concerning the side edge of the diced chip created by wet etching, it was observed that the chip size was reduced by about 50 μm from each side. In order to avoid the inside edge cutting the LED epilayer from the front side, the dimensions of the chip patterns were modified using the channel length from the backside of the CIC substrate. Chip patterns sized 1200 um × 1200 um with a channel length of 300 um could be matched to the front side of the LED epilayer sized 1140 um × 1140 um with a channel length of 360 μm during the dicing process. Details of the dimensions of the LED chips from the front side and those of the dicing chips from the backside of the CIC substrate are shown in Table 2.

**Table 2:** Dimensions of the LED chips from the front side and dicing patterns from the backside of the CIC substrate.

Patterns	Chip size	Channel width (μm)
LED chips from the front side	1140 μm × 1140 μm	360
Dicing chips from the backside	1200 μm × 1200 μm	300

**Figure 3:**
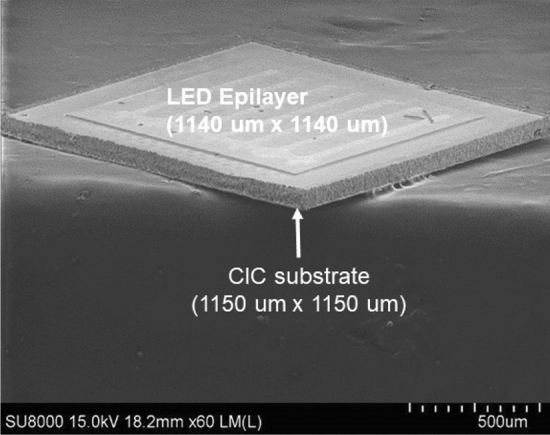
Typical single chip thin film AlGaInP LED on a CIC metal substrate.

**Figure 4:**
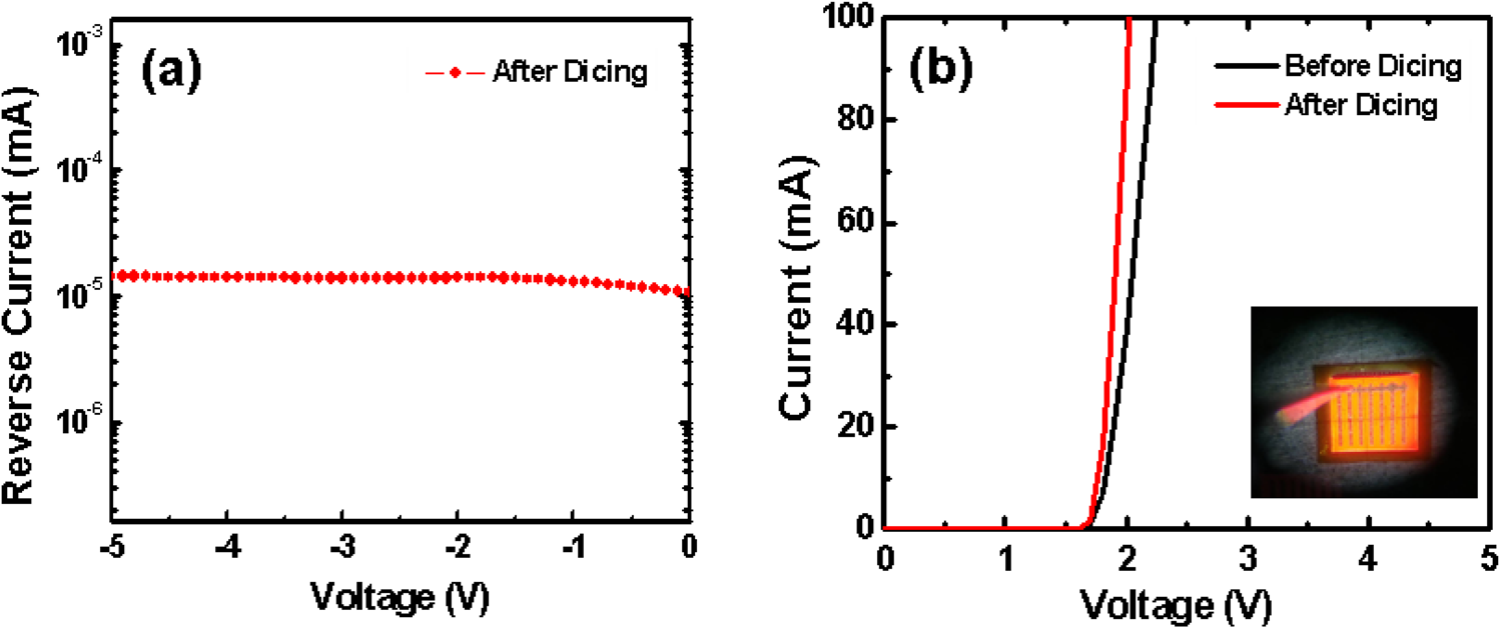
(**a**) Reverse I–V curves; (**b**) I–V characteristics of the LED/CIC chips before and after dicing. The inset shows the lightening picture of the LED/CIC chips after dicing.

Figure 3 presents the typical single chip thin-film AlGaInP LED on a CIC metal substrate. The dimensions of the LED epilayer were 1140 um × 1140 um and 1150 um × 1150 um for the diced chip. It is worthy to mention that the side wall of the LED and CIC substrate were very clean. There were no chipping or cracking problems which exist in dicing saw and ablation phenomenon resulting from the laser dicing. After dicing of the LED/CIC, the most important issue was the LED performance. The reverse and forward currents of the LED/CIC chips as a function of voltage are shown in Fig. 4a,b, respectively. The inset of Fig. 4b shows the turning on of the LED/CIC chips after dicing. A low leakage current of about 1.5 × 10^–8^ A was obtained when the reverse voltage was − 5 V. The forward voltages of the LED/CIC chips before chemical dicing were about 1.91 V and 2.81 V with an injection current of 20 mA and 350 mA, respectively. It is worthy to mention that although picosecond and femtosecond lasers are mature and remain a good candidate for dicing device chips, the leakage of LEDs is easily created by the metal splashing during the dicing. Furthermore, the CIC metal is easily melted back during dicing, especial for the small dicing channel. As concerning these problem, they did not occur for the chemical dicing.

**Figure 5:**
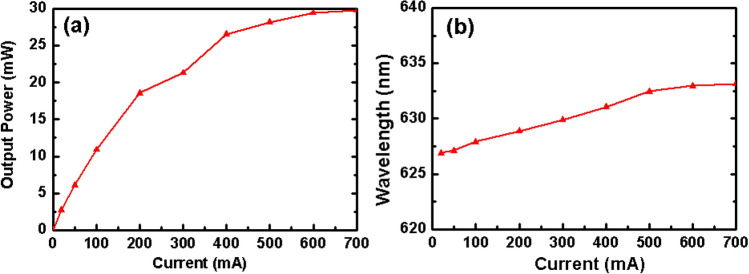
(**a**) Output power; (**b**) wavelength as a function of current for the LED/CIC chips after dicing.

After chemical dicing, the forward voltages of the LED/CIC chips were about 1.82 V and 2.34 V with an injection current of 20 mA and 350 mA, respectively. It was noted that the LED/CIC chips presented a lower forward voltage (1.82 V) due to the low electrical resistance and thickness of the substrate for the discrete chip^24^. Concerning the optical performance, the output power presented a linear relation with a current injection of 0 to 700 mA, as shown in Fig. 5a. Here, the output power became saturated as the injected current increased to 700 mA, due to the LED/CIC chips remaining packaged.

Moreover, the chips offered good thermal dissipation in the wavelength variation^8,24^. The thermal dissipation was related to the thickness of the substrates, meaning that a higher thermal dissipation required less thickness^8^. The wavelength of the LED/CIC chips changed from 627.9 to 633.13 nm as the current increased from 100 to 700 mA, as shown in Fig. 5b. These results suggested that the dicing of LED/CIC chips by the wet etching process could offer good electrical and optical properties.

## Conclusion

In this paper, dicing of LED/CIC chips was obtained by the wet etching process. The pattern of the substrate was made on the backside of the CIC substrate and compared with the front side of the AlGaInP LED chip pattern due to the side edge etching that occurred during the etching process. Due to its low cost, thickness, high thermal conductivity and ease of dicing, CIC substrate have the potential and mechanical strength to replace CuW and Si permanent substrates. Concerning the device performance, LED/CIC chips presented low leakage current, high output power and a low red shift phenomenon as they operated at a high injected current. The results suggested that the CIC substrate can be diced by the wet etching process without any physical damage or epilayer cracking, indicating that the wet etching process can be used in place of saw or laser dicing and extended for thin film micro LED applications, thereby meeting demands for next-generation device manufacturing processes.

## Methods

**Figure 6:**
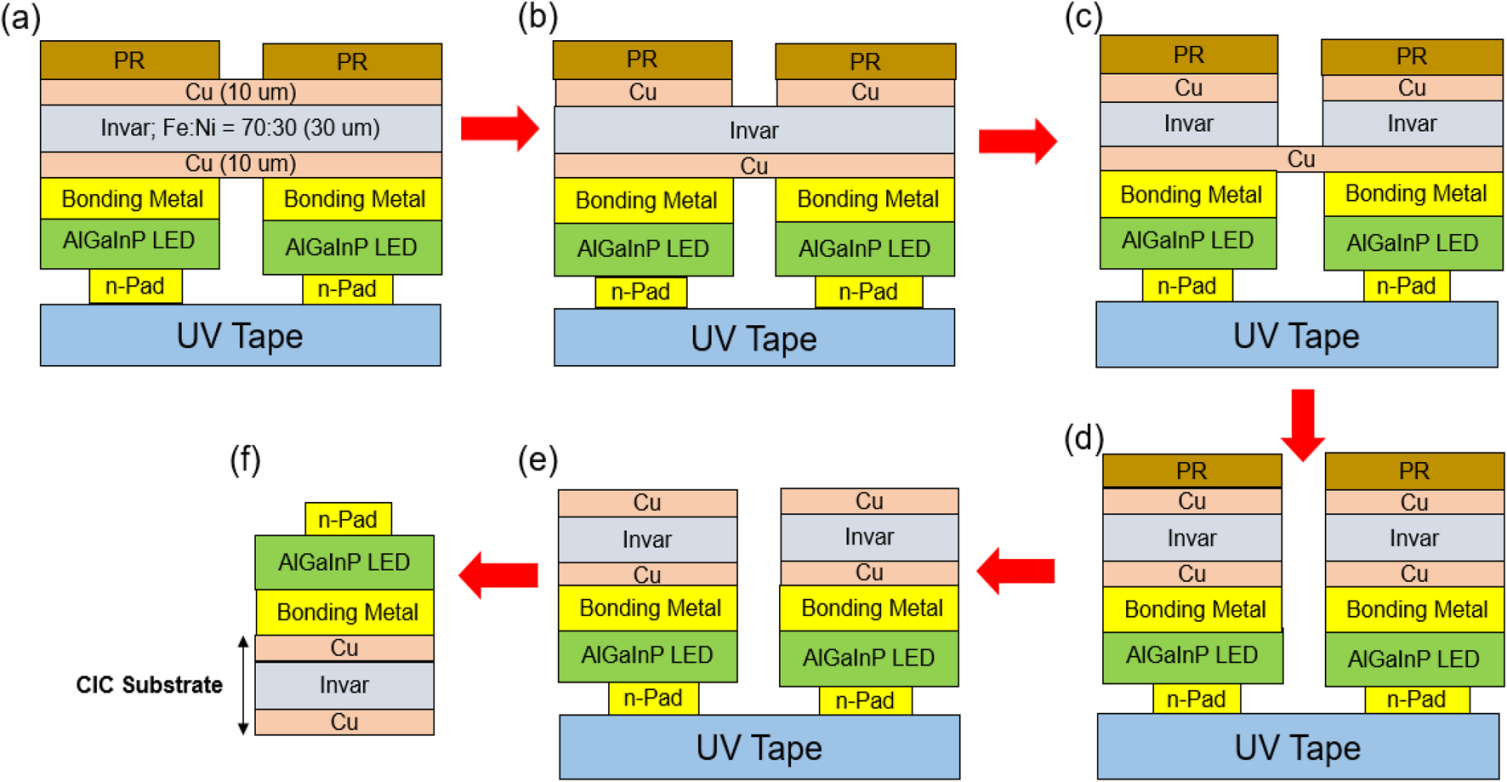
Process flow for the dicing of LED/CIC chips from the back side of the CIC.

**Figure 7:**
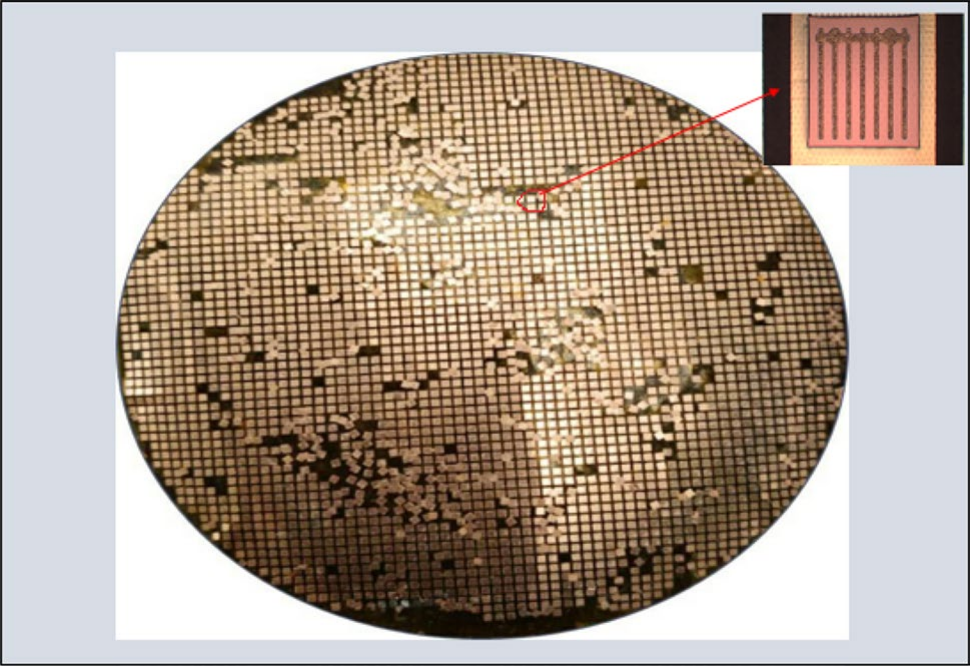
Diced LED/CIC chips from the back side of the CIC by the wet etching process.

The epilayer structure and fabrication process for thin film AlGaInP LED applications were proposed in our previous study^8,24,27^. Here, this study focused on the dicing of chips from the backside of the substrate by wet etching. Figure 6 shows the complete process flow chart for the dicing of the LED/CIC chips from the backside. First, the AlGaInP/LED chips on the CIC substrate was cleaned with acetone (ACE) and Isopropyl alcohol (IPA) to remove the impurities, then the pattern was created on the backside using a conventional lithography technology. After, the wafer was attached to UV tape from the front side to protect the AlGaInP/LED chips during the chemical dicing process, as shown in step (a). The 10-μm thick top layer (Cu) was removed from the Copper etcher chemical solution (CuR-8000S) in step (b), and the 30-μm thick middle layer (Invar) was removed from the Nickel etcher chemical solution (NiE-7520) in step (c). All etching process was carried out at room temperature and chemical was no stirring. Between the step (b) and step (c), wafer was rinsed by DI water. For the last Copper layer, the etching process was the same with the step b. After removing the entire CIC layers in the channel region, the AlGaInP/LED chips were diced from the backside of the substrate, as shown in Fig. 7. It can be find that the yield is about 90%. The 10% loss was resulted from the chips peeling off from the tape after the metal totally dicing. It can be overcome to find the high adhesive tape to increase the yield.

In this study, the dicing pattern from the backside was evaluated using the LED chip pattern from the front side of the substrate due to the existence of side edge etching during the etching process. LED/CIC chips could be successfully diced by the wet etching process without any physical damage or epilayer cracking, which can occur during saw or laser-based dicing. Wet etching dicing has the potential to dice substrates with thickness greater than 100 μm.

After completion of the whole process for the dicing of LED/CIC chips from the backside, the electrical and optical characteristics of the diced LED/CIC chips were measured using a multi-function power meter (KEITHLEY 2400) and an integrating sphere. The data for all measured LED/CIC chips were averaged from 20 different samples.
